# *MAL62* overexpression enhances uridine diphosphoglucose-dependent trehalose synthesis and glycerol metabolism for cryoprotection of baker’s yeast in lean dough

**DOI:** 10.1186/s12934-020-01454-6

**Published:** 2020-10-19

**Authors:** Xi Sun, Jun Zhang, Zhi-Hua Fan, Ping Xiao, Feng Li, Hai-Qing Liu, Wen-Bi Zhu

**Affiliations:** 1 College of Biological Engineering, Tianjin Agricultural University, Tianjin, 300384 People’s Republic of China; 2 Tianjin Engineering Research Center of Agricultural Products Processing, Tianjin, 300384 People’s Republic of China; 3 Frontier Science Center for Synthetic Biology and Key Laboratory of Systems Bioengineering (Ministry of Education), Tianjin University, Tianjin, 300072 People’s Republic of China; 4 Collaborative Innovation Center of Chemical Science and Engineering (Tianjin), School of Chemical Engineering and Technology, Tianjin University, Tianjin, 300072 People’s Republic of China; 5 Experiments and Teaching Center for Agricultural Analysis, Tianjin Agricultural University, Tianjin, 300384 People’s Republic of China

**Keywords:** *Saccharomyces cerevisiae*, Trehalose synthesis pathway, Alpha-glucosidase, Maltose, Glycerol, Freezing tolerant, RNA-seq, Lean dough

## Abstract

**Background:**

In *Saccharomyces cerevisiae*, alpha-glucosidase (maltase) is a key enzyme in maltose metabolism. In addition, the overexpression of the alpha-glucosidase-encoding gene *MAL62* has been shown to increase the freezing tolerance of yeast in lean dough. However, its cryoprotection mechanism is still not clear.

**Results:**

RNA sequencing (RNA-seq) revealed that *MAL62* overexpression increased uridine diphosphoglucose (UDPG)-dependent trehalose synthesis. The changes in transcript abundance were confirmed by quantitative reverse transcription–polymerase chain reaction (qRT-PCR) and enzyme activity assays. When the UDPG-dependent trehalose synthase activity was abolished, *MAL62* overexpression failed to promote the synthesis of intracellular trehalose. Moreover, in strains lacking trehalose synthesis, the cell viability in the late phase of prefermentation freezing coupled with *MAL62* overexpression was slightly reduced, which can be explained by the increase in the intracellular glycerol concentration. This result was consistent with the elevated transcription of glycerol synthesis pathway members.

**Conclusions:**

The increased freezing tolerance by *MAL62* overexpression is mainly achieved by the increased trehalose content via the UDPG-dependent pathway, and glycerol also plays an important role. These findings shed new light on the mechanism of yeast response to freezing in lean bread dough and can help to improve industrial yeast strains.

## Background

The use of frozen dough is now gradually emerging in a multitude of bakery and food chains due to its less time-consuming production after freezing and cheaper bake-off stations [1, 2]. However, freezing often causes oxidative stress and cell death to baker’s yeast [3], which reduces the yeast growth and gas production capacity [4, 5]. A number of protective molecules have been identified in yeast stress tolerance [6–8]. Among them, the disaccharide trehalose, which protects the cell membrane and stabilizes the protein structure, has captured wide attention [9]. Yeast trehalose is regulated by two major biosynthetic systems, system I and II. System I is uridine-5ʹ-diphosphoglucose (UDPG) dependent and contains several protein complexes, including one trehalose-6-phosphate synthase (encoded by *TPS1*) [10], one trehalose-6-phosphate phosphatase (encoded by *TPS2*), and one trehalose-synthesis protein complex (encoded by *TSL1*) [11]. The system II trehalose synthetic pathway is adenosine-diphosphoglucose (ADPG) dependent and uses maltose, a disaccharide, to synthesize trehalose [12, 13].

In baker’s yeast, the *MAL* gene family, which regulates maltose metabolism, consists of five multigene complexes, including *MAL1*, *MAL2*, *MAL3*, *MAL4*, and *MAL6*. Each gene complex encodes a maltose permease, an alpha-glucosidase, and a transacting MAL-activator [14]. We have shown previously that overexpression of *MAL62* enhances the cryotolerance of baker’s yeast [15] and speculated that multiple pathways may be involved in this phenomenon [16]. However, the mechanism for the enhanced freezing tolerance is still unknown.

To better understand the role of *MAL62* overexpression in the freezing tolerance of baker’s yeast in lean dough, and its possible mechanism, we used transcriptome analysis to characterize a *MAL62*-overexpressing strain and investigated the effects of overexpression of *MAL62* and deletion of the *TPS1* gene on maltose metabolism, trehalose and glycerol accumulation, and the freezing tolerance of baker’s yeast in lean dough.

## Materials and methods

### Strains, plasmids, and growth conditions

The yeast and bacterial strains as well as the plasmids used in this study are listed in Table 1. The parent industrial strain BY14 was used to create the high-leavening haploid BY14a strain, which was used to create all of the other strains, including the overexpression and deletion strains.

**Table 1:** Characteristics of the strains used in the present study

Strains or plasmids	Relevant characteristics	Reference or source
Strains		
* E. coli* DH5α	Φ80 *lacZ* M15*ΔlacU169Δ recA1 endA1 hsdR17 supE44 thi-1 gyrA relA1*	YCC
BY14a^a^	*MATa*	YCC
BY14a + K	*MATa*, Yep-K	[15]
B + MAL62	*MATa*, Yep-PMK	[15]
B-T	*MATa*, *tps1Δ*:: *KanMX*	This study
B-T + M	*MATa*, *tps1Δ*:: *MAL62*	This study
B + TPS1	*MATa*, Yep-PTK	[16]
Plasmids		
pUG6	*E. coli/S. cerevisiae* shuttle vector, containing *Amp*^+^, *loxP-kanMX-loxP* disruption cassette	[31]
pPGK1	*bla LEU2 PGK1*_*P*_*-PGK1*_*T*_	[58]

YCC: Yeast Collection Center of the Tianjin Key Laboratory of Industrial Microbiology

^a^BY14a was selected as a high-leavening capacity haploid from 32 clones derived from BY14 (data not shown)

The *Escherichia coli DH5a* and yeast strains were cultured as described previously [15]. Briefly, yeast cells in yeast extract peptone dextrose (YPD) medium (1% yeast extract, 2% peptone, and 2% dextrose) were cultured at 30 ℃ on a rotor with a speed of 180 rpm. G418 (final concentration of 0.08%) (Thermo Fisher, Waltham, MA, USA) was added to YPD plates to select G418-resistant transformants. After growing in YPD for 24 h, cultured cells (20 mL) were inoculated into 200 mL of cane molasses medium (0.5% yeast extract, 0.05% (NH_4_)_2_SO_4_, and 12° Brix cane molasses), giving an initial OD_600_ value of 0.4. The cells were cultured at 30 °C to an OD_600_ value of 1.8 (about 24 h), then centrifuged at 4 °C and 5000 rpm for 5 min, and finally washed twice with sterile water. A modified low sugar model liquid dough (LSMLD) medium [17] was mainly used for the measurements of trehalose, intracellular glycerol, and the cell viability during prefermentation and after prefermentation freezing.

### RNA sequencing (RNA-seq)

RNA-seq-based transcriptome analysis was performed to identify the differentially expressed genes after *MAL62* overexpression. Cells constitutively overexpressing *MAL62* (B + MAL62) and the control (BY14a + K) were grown in cane molasses medium. RNA isolation and cDNA synthesis were performed as previously described [18]. Briefly, total RNA was isolated using the hot acid phenol method, followed by DNase treatment. The RNA concentration was measured using a Qubit fluorometer and a Qubit RNA Assay Kit (Thermo Fisher Scientific, Waltham, MA, USA). The RNA integrity was assessed using a Bioanalyzer 2100 system (Agilent Technologies, Santa Clara, CA, USA) and an RNA Nano 6000 Assay Kit.

The RNA-seq libraries were generated from 1 μg of RNA from each sample using a NEBNext Ultra RNA Library Prep Kit (New England Biolabs, Ipswich, MA, USA), according to the manufacturer’s instructions. The clustering of samples, which was index coded to attribute individual sample sequences, was performed using a cBot Cluster Generation System (Illumina, San Diego, CA, USA). The RNA libraries were sequenced using an Illumina Hiseq 2500 system (Illumina). Paired-end reads of 125 bp/150 bp were generated and analyzed.

### RNA-seq data analysis

The differential expression of two different groups was analyzed using DESeq R software (https://www.bioconductor.org, version 1.18.0). Genes with a false-discovery rate-adjusted *p*-value < 0.05 were considered as differentially expressed.

Volcano plots and hierarchical clustering were used to screen the differentially expressed genes and to analyze the clusters of differentially expressed genes. GOseq [19] was used for gene ontology (GO) term enrichment analysis. The Kyoto Encyclopedia of Genes and Genomes (KEGG) was used to determine the pathways with upregulated genes. STRING (https://www.string-db.org/) and Cytoscape (https://cytoscape.org) software programs were used for protein–protein interaction (PPI) network analysis.

### Validation of gene expression levels

Quantitative reverse transcription–polymerase chain reaction (qRT-PCR) was used to detect the expression levels of the target genes. qRT-PCR was conducted using the THUNDERBIRD probe one-step qRT-PCR kit (TOYOBO, Osaka, Japan). The yeast *UBC6* gene, which encodes a ubiquitin-conjugating enzyme involved in endoplasmic reticulum-resident proteins for degradation, was used as a reference gene [20]. The PCR primers are listed in Table 2. Yeast cDNA was extracted using an RNAiso kit (Takara Biotech, Dalian, China) and a PrimeScript RT reagent kit with gDNA eraser (Perfect Real Time, Takara Biotech). The PCR was conducted using a CFX96 real-time PCR system (Bio-Rad, Hercules, CA, USA). The reaction conditions were as follows: 95 ℃ for 30 s; 61 °C for 20 min; 95 °C for 30 s; 43 cycles of 95 °C for 5 s, 55 °C for 10 s, and 74 °C for 15 s; and 72 °C for 5 min. Quantitative analysis of the qRT-PCR was conducted using the 2^−ΔΔCT^ method.

**Table 2:** Primers used in this study

Name	5′ → 3′ DNA sequences
For recombinant construction	
TU1-F	GATGCTGTTGTTCTTTCTTCTGTTT
TU1-R	CCTGCAGCGTACGAAGCTTCAGCTGAGTTCTATGTCTTAATAAGTCTGTA
KAN1-F	TACAGACTTATTAAGACATAGAACTCAGCTGAAGCTTCGTACGCTGCAGG
KAN1-R	GATCGTCTCATTTGCATCGGGTTCAGCATAGGCCACTAGTGGATCTGATA
TD1-F	TATCAGATCCACTAGTGGCCTATGCTGAACCCGATGCAAATGAGACGATC
TD1-R	ACTTTCTAAAATGGCTATATAGGGG
TU2-R	TTCAGTTTTGGATAGATCAGTTAGAAGTTCTATGTCTTAATAAGTCTGTA
PGKP-F	TACAGACTTATTAAGACATAGAACTTCTAACTGATCTATCCAAAACTGAA
PGKP-R	TTTCTGGATGATCAGAAATAGTCATGTTTTATATTTGTTGTAAAAAGTAG
MAL-F	CTACTTTTTACAACAAATATAAAACATGACTATTTCTGATCATCCAGAAA
MAL-R	AGAAAAGAAAAAAATTGATCTATCGTTATTTGACGAGGTAGATTCTACCT
PGKT-F	AGGTAGAATCTACCTCGTCAAATAACGATAGATCAATTTTTTTCTTTTCT
PGKT-R	CCTGCAGCGTACGAAGCTTCAGCTGTAACGAACGCAGAATTTTCGAGTTA
KAN2-F	TAACTCGAAAATTCTGCGTTCGTTACAGCTGAAGCTTCGTACGCTGCAGG
For PCR verification	
UUK-F	ATCTAAGAGGACGGTTGCTG
UUK-R	GTCAAGACTGTCAAGGAGGG
KDD1-F	TCGCAGACCGATACCAGGAT
KDD1-R	TCAACGGATGGGAAAGCAAT
UUP-F	GCGGTCCGTTCTGTGGTT
UUP-R	CCCTCTGTGGCGGTCTAT
PPM-F	CACATGCTATGATGCCCACT
PPM-R	CGCAAACAAACGGAGGTA
MPT-F	CGAAAGATAAGCCCAATG
MPT-R	CTGTAACGAACGCAGAAT
PTK-F	AAATTCTGCGTTCGTTAC
PTK-R	CCGTCAGCCAGTTTAGTC
KDD2-F	TATGTGAATGCTGGTCGCTAT
KDD2-R	CCGTTGCTACTGCCGTTA
For RT-qPCR	
qGDB1-F	AGCCTAACTTCGGCACTC
qGDB1-R	CACCGTCATCTAATCTCAAATA
qEMI2-F	GGCAAGGATGTCGTGAGGTT
qEMI2-R	AGCCTGAAGTGTAGCAGTGG
qGLK1-F	ATCACGAAGTTGCCACAG
qGLK1-R	TCACCCAAGAACATCCCT
qHXK2-F	TCCGTTTACAACAGATACCC
qHXK2-R	ATAACAGCGGCACCAGCA
qHXK1-F	GTGTCAAGACCACTCTGCCA
qHXK1-R	GGATCTTTGCTTGCGTCACC
qPGM2-F	GAAAAGGACGGTGTTTGGGC
qPGM2-R	GGCTGGGAAGGCGGAATTAA
qPRM15-F	TAAGCAAGACCGCAACCCAA
qPRM15-R	CCAATCCCTGAGACGCTTGT
qUGP1-F	CGAGAGCAACACAAACAGCG
qUGP1-R	CCGGGTTGGGAGACTTGATC
qTPS1-F	GGGGCAAGGTTGTTCTG
qTPS1-R	TCACGGGTGGACGAGAC
qTPS2-F	CCACCACTGCCCAAGACAAT
qTPS2-R	CAGGTTGCGTTCGGTTCTTG
qTPS3-F	TGCTCCGTCTGCTAGAGTCT
qTPS3-R	GGATCGACATCTGGAACGCT
qUBC6-F [59]	GGACCTGCGGATACTCCTTAC
qUBC6-R [59]	TAATCGTGTGTTGGGCTTGA

### Measurement of enzymatic activities

Cells were grown in cane molasses medium to the late-log phase, and the Tps1 activity was measured as previously described [21]. One unit of Tps1 activity was defined as the production of 1.0 μM of trehalose-6-phosphate per minute. The final activity was calculated based on the cell dry weight (CDW). Data were expressed as the mean ± standard deviation (SD) from three independent experiments.

For α-glucosidase activity determination, cells were grown in cane molasses medium to an OD_600_ value of 1.8, then inoculated into LSMLD medium, and cultivated for 2.5 h. Crude extracts were prepared using the Salema-Oom method [22], and the α-glucosidase activity was measured as previously described [23]. Data were expressed as the mean ± SD from three independent experiments.

To determine the activity of other enzymes, including hexokinase, phophoglucomutase, UGPase, and glycerol-3-phosphate dehydrogenase (G3PDH), cells were grown in cane molasses medium to the late-log phase, centrifuged at 5000 rpm and 4 °C for 5 min, and then washed twice with cold sterile water. The activities of hexokinase [24], phosphoglucomutase [25], UGPase [26], and G3PDH [27] were assayed as described previously. The protein concentration was measured using the Bio-Rad protein assay kit (Bio-Rad, Richmond, USA), according to the manufacturer’s instructions. Data were expressed as the mean ± SD from three independent experiments.

### Measurement of intracellular trehalose contents

Cells were grown in cane molasses medium to the late-log phase for the trehalose accumulation experiments. For the trehalose degradation tests during prefermentation, cells were grown in LSMLD medium for 25 min. Freshly cultured cells (0.1 g) were washed twice with water. Trehalose was extracted with 4 mL of 0.5 M trichloroacetic acid. Trehalose in the extract was estimated by the method outlined by Stewart [28] as well as with the anthrone reagent described by Spiro [29]. Data were expressed as the mean ± SD from three independent experiments.

### Measurement of extracellular maltose

For the measurement of extracellular maltose, cells were grown in LSMLD medium for 4 h. Cultured cells were filtered through a 0.45-µm-pore-size cellulose acetate filter (Millipore, Danvers, MA, USA). The extracellular maltose was measured by high-pressure liquid chromatography (HPLC) analysis using an Aminex HPX-87H column (Bio-Rad, Hercules, CA, USA) and an HPLC pump (Waters 515). The column was eluted at 65 °C with 5 mM H_2_SO_4_ at a flow rate of 0.6 mL/min [30]. Maltose was detected with a differential refractometer detector (Waters 410 RI). Data were expressed as the mean ± SD from three independent experiments.

### Yeast strain construction

Yeast genomic DNA was extracted using a yeast DNA isolation kit (Omega Bio-Tek, Norcross, GA, USA). The *tps1Δ* (B-T) strain (Table 1) was constructed as follows: the TPS1U fragment containing the *TPS1* upstream homologue sequence and the TPS1D fragment containing the *TPS1* downstream homologue sequence were amplified from the BY14a yeast genome with the primers TU1-F/TU1-R and TD1-F/TD1-R, respectively. The fragment *KanMX* was amplified from the plasmid pUG6 using the primers KAN1-F/KAN1-R [31]. Then, the fragments of TPS1U, TPS1D, and *loxP-KanMX-loxP* were transferred into BY14a using the lithium acetate/polyethylene glycol method [25]. G418 (300 μg/mL) was used to select the positive recombinants, which were further verified by PCR with the primers UUK-F/UUK-R and KDD1-F/KDD1-R. The *tps1Δ *plus *MAL62*-overexpression (B-T + M) strain (Table 1) was constructed as follows: The *MAL62* gene was amplified from the BY14a genome with the primers TU1-F/TU2-R, TD1-F/TD1-R, and MAL-F/MAL-R. The fragments containing the yeast phosphoglycerate kinase gene promoter (*PGK1p*) and terminator (*PGK1t*) were amplified from the BY14a genome with the primers PGKP-F/PGKP-R and PGKT-F/PGKT-R, respectively. The fragment *loxP-KanMX-loxP* was amplified from the plasmid pUG6 using the primer pair KAN2-F/KAN1-R. Six fragments (TPS1U, *PGK1*_*P*_, *MAL62*, *PGK1*_*T*_, *KanMX*, and TPS1D) were transferred into the BY14a strain, and the recombinant B-T + M strain was verified via PCR using the primer pairs of UUP-F/UUP-R, PPM-F/PPM-R, MPT-F/MPT-R, PTK-F/PTK-R, and KDD2-F/KDD2-R. B + MAL62, the *MAL62-*overexpression strain, was constructed as described previously [15].

### Measurement of intracellular glycerol content

To measure the intracellular glycerol levels, cells were cultured in cane molasses medium for 24 h at 30 °C and then transferred to LSMLD medium. The cells were prefermented for 5, 10, 15, 20, and 25 min. Approximately 25 mg (wet weight) of cells was washed, resuspended in 1 mL of deionized water, and boiled twice (30 min, with occasional shaking). The supernatants were then centrifuged for 10 min at 15,000×*g*. The level of glycerol was measured as described previously [32]. Data were expressed as the mean ± SD from three independent measurements.

### Measurement of cell viability and leavening ability

Yeast cells were cultured in cane molasses medium [5 g/L yeast extract, 0.5 g/L (NH_4_)_2_SO_4_, and 12° Brix cane molasses] for 24 h at 30 °C and then transferred to LSMLD medium. The cells were prefermented for 5, 10, 15, 20, and 25 min, and then moved to a − 20 °C freezer for 7 days. The cell viability was measured after freezing, as described previously [15]. Data were expressed as the mean ± SD from three independent experiments.

The leavening abilities were measured by the amount of carbon dioxide (CO_2_) produced by the lean dough. The lean dough contained 280 g of standard flour, 4 g of salt, 9 g of fresh yeast, and 150 mL of water. The dough was mixed at 30 °C for 5 min and divided into 50-g pieces. The dough pieces were placed in a fermentograph (Type JM 451; Mekab Försäljnings AB, Nässjö, Sweden). The production of CO_2_ was measured at 30 °C for 2 h. To examine the effect of freezing and thawing on the leavening ability, the dough was frozen for 1 to 4 weeks at − 20 °C and then thawed for 30 min at 30 °C. The production of CO_2_ was measured while the dough was at 30 °C for 2 h. The measurement was repeated three times, and data were expressed as the mean ± SD.

### Statistical analysis

Data were represented as the mean ± SD from three independent experiments. Differences among the various strains were analyzed using analysis of variance. Differences between the parent and the *MAL62*-overexpression strains were analyzed using the Student’s *t*-test. For all analyses, *p* < 0.05 was considered statistically significant.

## Results

### *MAL62* overexpression enhances the UDPG-dependent trehalose synthesis

**Fig. 1:**
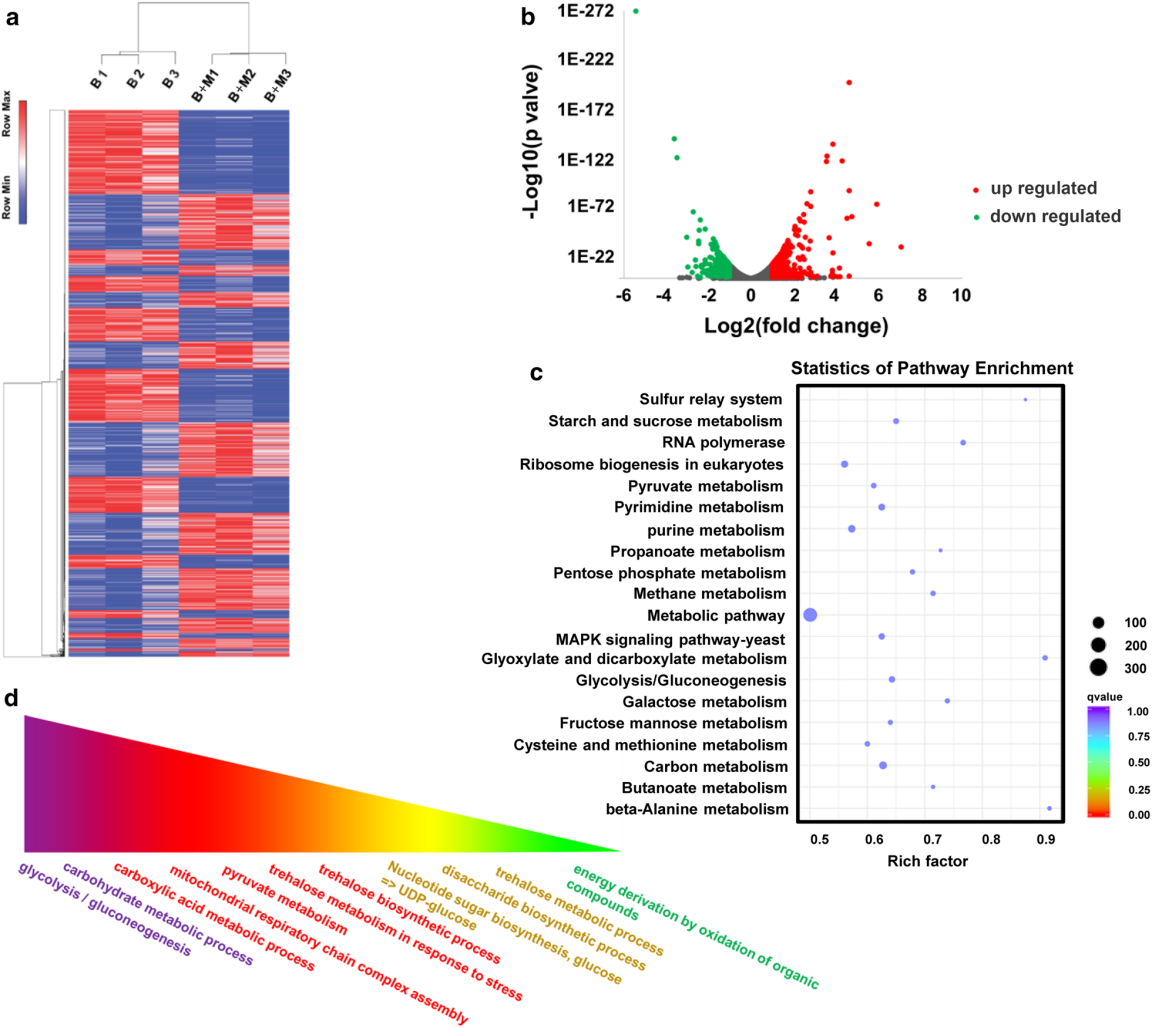
Results of RNA-Seq transcriptome analysis. **a** Hierarchical clustering of the significant genes (B indicates BY14a + K, B + M indicates B + MAL62). **b** Volcano plot of all genes. The downregulated genes are shown in green, and the upregulated genes are shown in red. **c** Kyoto Encyclopedia of Genes and Genomes analyses. **d** Gene ontology functional enrichment analyses. The functions are arranged from deep to shallow according to their relevance

**Fig. 2:**
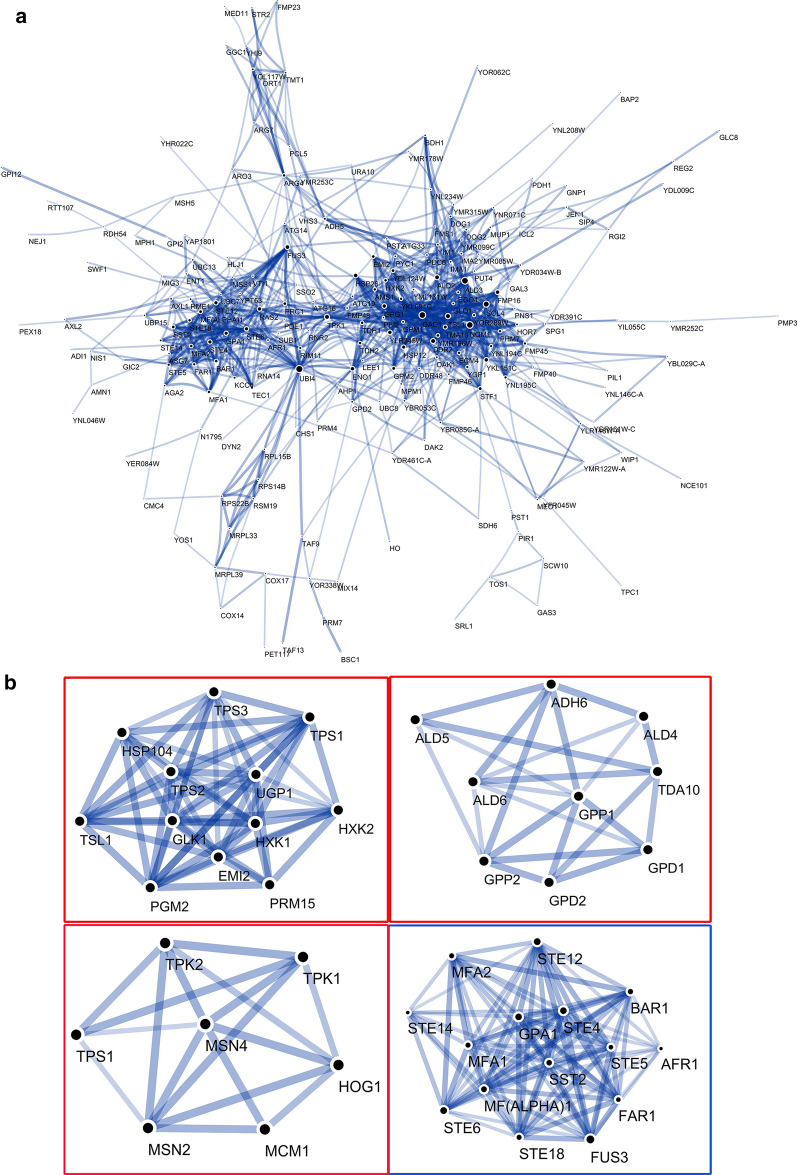
Interactions between the upregulated proteins. **a** Protein–protein interaction networks. **b** Significant modules in the protein–protein interaction network

RNA-seq analysis was performed to identify the differentially expression genes when *MAL62* was overexpressed. The results showed that *MAL62* overexpression caused significant changes in gene expression (Fig. 1a). Compared to the control (BY14a + K), 1460 genes were downregulated (Fig. 1b, green) and 1506 genes were upregulated (Fig. 1b, red). KEGG analysis revealed that the upregulated genes were mainly enriched in the metabolism and synthesis of carbohydrates (Fig. 1c). GO analysis of the biological processes (molecular function, cellular component, and biological process) showed that several processes involving trehalose were affected by *MAL62* overexpression (Fig. 1d). In addition, STRING analysis demonstrated that *MAL62* overexpression caused upregulation of *TPS1*, *TPS2*, *TPS3*, and *UGP1*, which are all key genes of the UDPG pathway (Fig. 2 and Additional file 1: Figure S1).

**Fig. 3:**
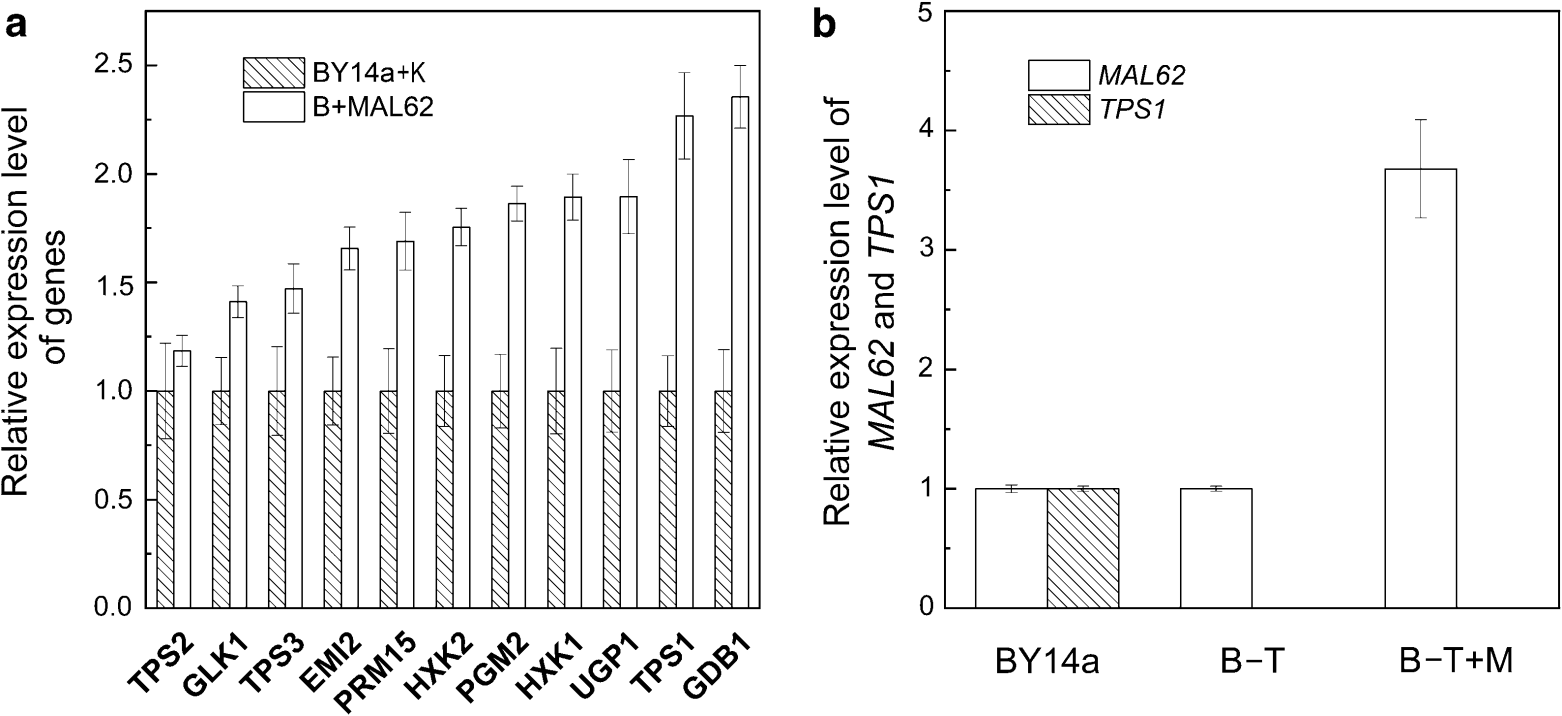
Quantitative RT-PCR analysis of the relative expression levels of genes in the recombinant strain B + MAL62 and the control strain BY14a + K (**a**) as well as in the recombinant strains B-T and B-T + M and the control strain BY14a (**b**). Data are expressed as the mean ± SD (indicated as error bars) of three independent experiments

To further examine the possible involvement of the UDPG pathway on trehalose synthesis, the expression levels of trehalose metabolism-related genes (*GLK1*, *EMI2*, *HXK1*, *HXK2*, *PGM2*, *PRM15*, *UGP1*, *GDB1*, *TPS1*, *TPS2*, and *TPS3*) were analyzed by qRT-PCR. Our results showed that all of these genes, except for *TPS2*, had a significantly higher expression in the B + MAL62 strain than in the BY14a + K strain (Fig. 3a). The fold changes were as follows: *GDB1*, 2.36; *TPS1*, 2.27; *UGP1*, 1.90; *HXK1*, 1.89; *EMI2*, 1.66; *PRM15*, 1.69; *HXK2*, 1.75; *PGM2*, 1.86; *GLK1*, 1.41; and *TPS3*, 1.47. No significant change of *TPS2* was observed between the B + MAL62 and BY14a + K strains. The enzyme activity measurements showed that all of the tested key enzymes related to the UDPG pathway, except for hexokinase, were of higher activity in the B + MAL62 strain than in the BY14a + K strain (Table 3).

**Table 3:** Activity of enzymes related to the UDPG pathway and glycerol metabolism

	BY14a + K	B + MAL62
Hexokinase (U/mg pro)	1.19 ± 0.09	1.30 ± 0.11
Phosphoglucomutase (U/mg pro)	0.14 ± 0.01	0.31 ± 0.03*
UGPase (U/mg pro)	0.43 ± 0.06	1.10 ± 0.08*
Tps1 (U/g CDW)	0.79 ± 0.07	1.20 ± 0.05*
Glycerol-3-phosphate dehydrogenase (U/mg pro)	1.5 ± 0.07	2.83 ± 0.21*

Data are expressed as the mean ± SD from three independent experiments

*CDW* cell dry weight

^*^P < 0.05 in comparison with the parent strain

**Fig. 4:**
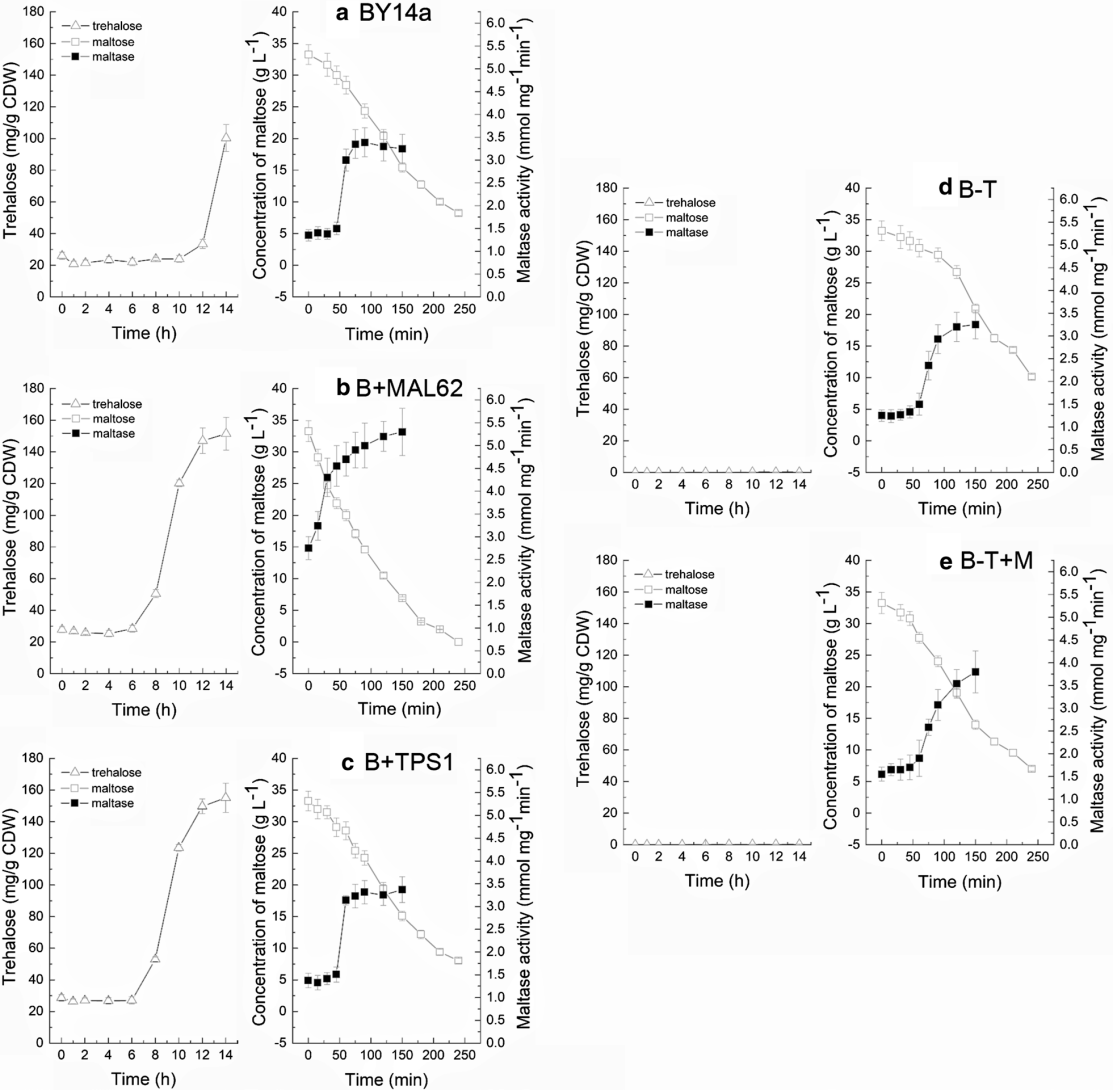
Measurements of alpha-glucosidase (maltase) activity, residual maltose concentration, and intracellular trehalose content in five yeast strains: BY14a (**a**), B + MAL62 (**b**), B + TPS1 (**c**), B-T (**d**), and B-T + M (**e**)

**Fig. 5:**
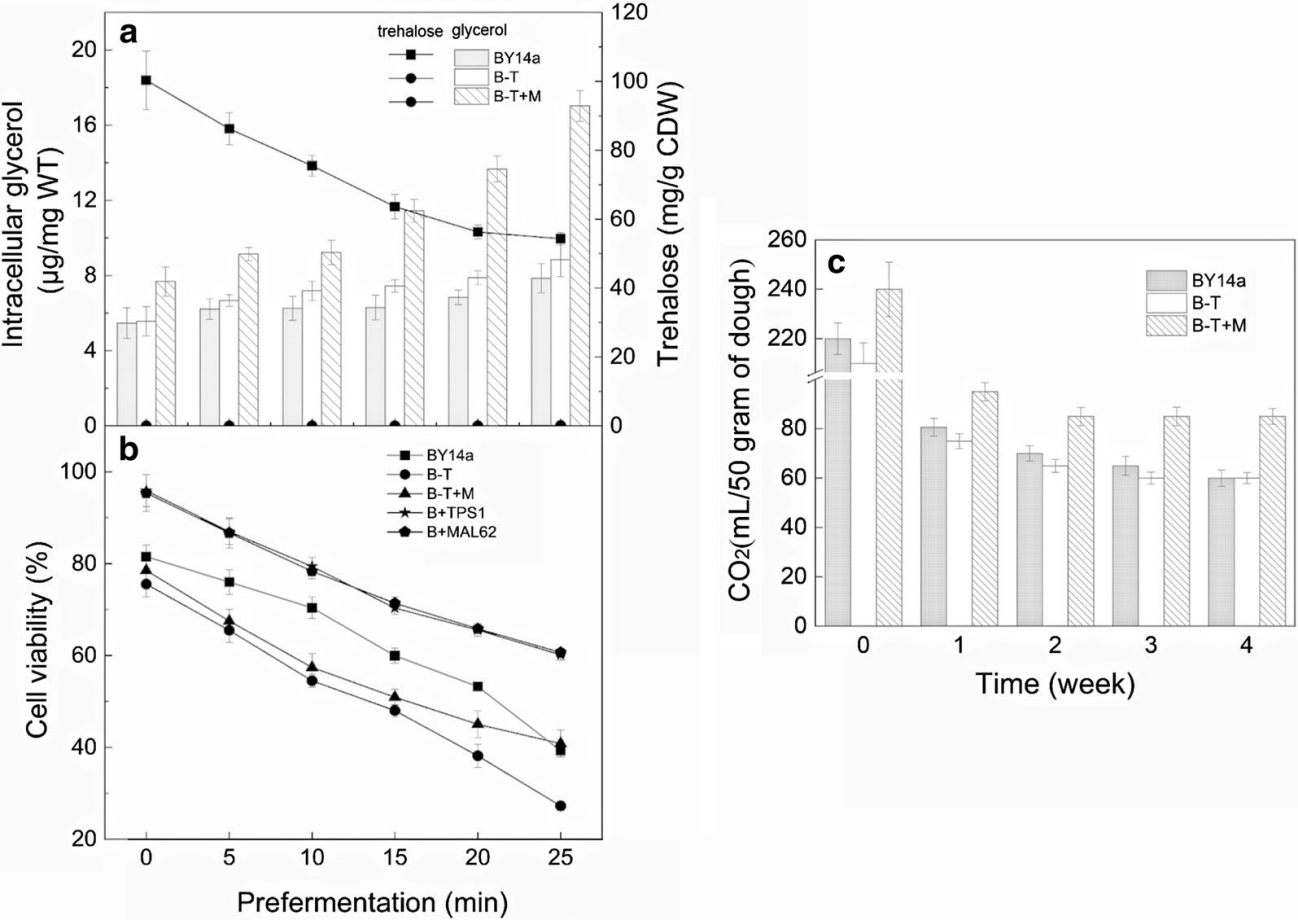
Contents of intracellular glycerol and trehalose (**a**) and cell viability (**b**) after different fermentation times (0–25 min) and freezing in LSMLD medium for 7 days. Measurement of yeast CO_2_ production in the lean dough after different periods of freezing (0–4 weeks) (**c**). Data are expressed as the mean ± SD (indicated as error bars) of three independent experiments

Disruption of the *TPS1* gene diminishes the UDPG-dependent trehalose synthase activity [13]. To further understand the role of *MAL62* overexpression in trehalose synthesis, we constructed a *tps1Δ* strain (B-T), and *MAL62* was overexpressed in a *tps1Δ* strain (B-T + M) to eliminate the effects of the UDPG-dependent trehalose synthesis pathway. Figure 3b shows that the expression levels of *TPS1* in the B-T and B-T + M strains were not detectable. Compared with the maltose fermentation (a maltose concentration decrease of 39.86%) and alpha-glucosidase activity (3.91 mmol mg^−1^ min^−1^, on average) of B + MAL62, B-T and B-T + M had lower consumption of maltose (a maltose concentration decrease of only 6.07%, on average) and lower activities of alpha-glucosidase (just maintained at 1.46 mmol mg^−1^ min^−1^, on average) in the first 60 min of fermentation (Fig. 4). One possible cause of this is the slower growth rates of the two *tps1Δ* strains [33]. However, it is worth noting that even when the alpha-glucosidase activities started to rise at 60 min and reached the peak at nearly 150 min during fermentation, the trehalose levels in the B-T and B-T + M strains were still not detectable These results suggest that *MAL62* overexpression activates the UDPG pathway, which then causes the accumulation of intracellular trehalose and enhanced cryotolerance. To further confirm this finding, we overexpressed *TPS1* (B + TPS1). The consumption of maltose and the activity of alpha-glucosidase of B + TPS1 were similar to those of BY14a and B-T (Fig. 4). However, the trehalose accumulation (Fig. 4b, c) and cell viability (Fig. 5b) remained at the same level as those of B + MAL62, further suggesting that *TPS1* plays an important role in cryoprotection.

### *MAL62* overexpression enhances glycerol metabolism

RNA-seq and STRING analyses showed that *MAL62* overexpression caused upregulation of *GPD1*, *GPD 2*, *GPP1*, and *GPP2* (Fig. 2, Additional file 2: Figure S2, and, Additional file 3: Figure S3). *GPD1* and *GPD 2* encode the rate-limiting enzymes in the high-osmolarity glycerol mitogen-activated protein kinase (HOG-MAPK) pathway, which induces glycerol accumulation [34–37]. We assayed the activity of glycerol-3-phosphate dehydrogenase in the BY14a + K and B + MAL62 strains. As shown in Table 3, the activity of glycerol-3-phosphate dehydrogenase in B + MAL62 was 88.7% higher than that in BY14a + K, suggesting that *MAL62* overexpression causes an increase in the glycerol content.

To further determine the change of glycerol content and its possible cryopreservation effect in the *MAL62*-overexpressed strain, we used B-T and B-T + M to eliminate the effects of trehalose. As shown in Fig. 5a, the B-T strain exhibited a similar intracellular glycerol synthesis rate compared to the BY14a strain, suggesting that the deletion of *TPS1* did not affect glycerol synthesis. However, the intracellular glycerol content in the B-T + M strain was significantly higher than that in the BY14a or B-T strain after prefermentation for 15 min and freezing for 7 days (*p* < 0.05). After prefermentation for 25 min and freezing for 7 days, the intracellular glycerol content in the B-T + M strain increased by approximately 121.5%, compared with prefermentation for 0 min, while the intracellular glycerol contents in the BY14a and B-T strains increased by only 23.9% and 32.8%, respectively. These results suggest that *MAL62* overexpression positively correlates with the accumulation of intracellular glycerol.

Next, we examined whether the increased glycerol level by *MAL62* overexpression affects the freezing tolerance by measuring the cell viability. As shown in Fig. 5b, a longer prefermentation duration caused a significant decrease in the cell viability after freezing for 7 days in all three strains (BY14a, B-T, and B-T + M). After prefermentation for 25 min, the cell viability of the BY14a and B-T + M strains was similar, but the cell viability of the B-T strain was significantly lower than those of the BY14a and B-T + M strains (*p* < 0.05). Compared to the BY14a and B-T strains, the cell viability of the B-T + M strain showed a minor decrease after prefermentation for 15 min (Fig. 5b). These findings suggest that in addition to triggering the accumulation of trehalose, *MAL62* overexpression also causes an increment in the glycerol content, which can enhance freezing tolerance.

### Increased glycerol content by *MAL62* overexpression enhanced the leavening ability after long-term freezing

The possible effect of the increased glycerol level by *MAL62* overexpression on the leavening ability after long-term freezing was determined by measuring CO_2_ production. As shown in Fig. 5c, the CO_2_ production in all strains decreased as the freezing time increased from 1 to 4 weeks. However, the CO_2_ production of the B-T + M strain was significantly higher than either the BY14a or B-T strain before freezing (time = 0) and after freezing for 1 to 4 weeks (*p* < 0.05). These results suggest that the increased glycerol content by *MAL62* overexpression can mitigate the loss of the leavening ability after exposure to the stress induced by long-term freezing.

## Discussion

Our comparative transcriptome analysis revealed that overexpression of *MAL62* causes significant differences in gene expression, as compared to its wild-type control (BY14a + K). Many of these genes are involved in the stress response, especially freezing stress pathways. Several genes involved in starch and sucrose metabolism, glycerophospholipid metabolism, and glycerolipid metabolism are also differentially expressed between these two strains. KEGG analysis further confirmed that many of the pathways are involved in cryotolerance of the B + MAL62 strain. In baker’s yeast, trehalose is believed to be the primary compound affecting the viability of yeast in frozen dough [38, 39]. Using mutants of *S. cerevisiae* deficient in trehalose synthesis, degradation, or transport, studies have shown that trehalose can protect cells exposed to freezing and dehydration [40–42]. We have previously reported that the enhanced freezing tolerance by *MAL62* overexpression is related to the increased activity of Tps1 [16]. Our current study provides further evidence that genes involved in starch and sucrose metabolism, including *GLK1*, *EMI2*, *HXK1*, *HXK2*, *PGM2*, *PRM15*, and *UGP1*, had a higher expression level in the B + MAL62 strain (Fig. 3a and Additional file 1: Figure S1). In addition, PPI network analysis revealed a high score for Tps1, Tps2, and Tps3 (Fig. 2b). The enzyme activities, metabolism of trehalose and glycerol, cell viability, and gas production after freezing provided further confidence (Table 3 and Figs. 4, 5a, b) that the enhanced freezing tolerance by *MAL62* overexpression is related to the UDPG-dependent trehalose synthesis pathway. However, *MAL62* is an enzyme, not a transcription factor. It is not clear how its overexpression affects the expression of so many genes. One possibility is that *MAL62* may affect the expression of some transcription factors due to the fact that the *MAL6* locus has been reported to be located in nuclei [43, 44].

Microorganisms often accumulate different solutes, such as ions, amino acids, and polyols, to mitigate water loss [45, 46] when they face a water shortage. Baker’s yeast responds to freeze stress-induced hyperosmotic stress by activating the HOG-MAPK pathway, which induces glycerol accumulation [34–37]. It has been extensively documented that glycerol 3-phosphate dehydrogenase, which is encoded by *GPD1* and *GPD2*, is the key enzyme in the production of glycerol [47, 48]. The strong upregulation of *GPD1* under hyperosmotic stress [49, 50] is at least partly controlled by the HOG1-MAPK cascade [49, 51]. Our transcriptome analysis revealed that HOG1, Msn2, and Msn4 scored high on the PPI network (Fig. 2b). Genes and rate-limiting enzyme activity (glycerol-3-phosphate dehydrogenase) involved in glycerol biosynthesis were also upregulated in the B + MAL62 strain (Additional file 2: Figure S2 and Additional file 3: Figure S3, and Table 3), suggesting that *MAL62* overexpression induces the accumulation of glycerol through the HOG pathway.

**Fig. 6:**
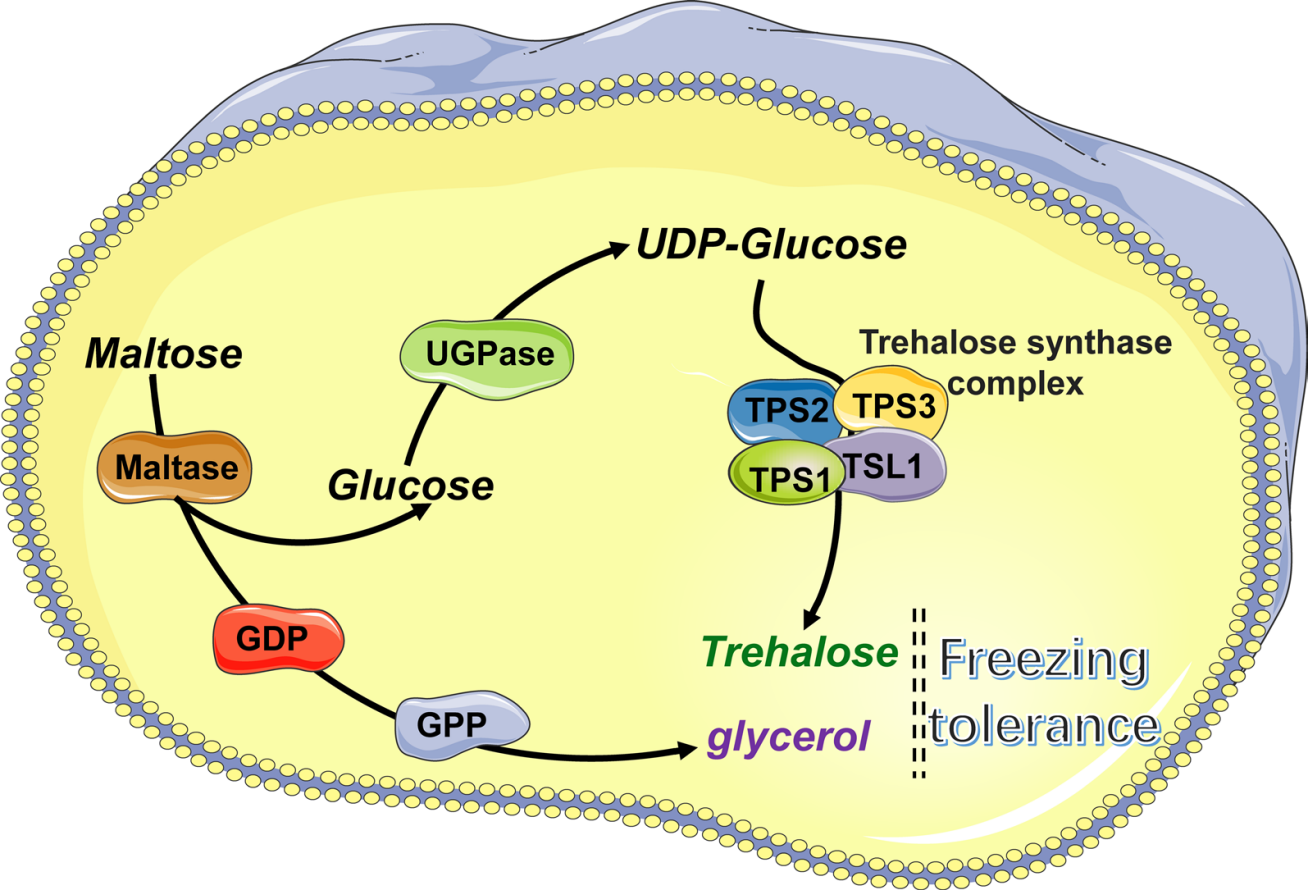
Proposed regulatory mechanism of *MAL62* overexpression in yeast. *MAL62* overexpression induces the UDPG-dependent trehalose synthesis pathway and the HOG-MAPK-dependent glycerol synthesis pathway

It has been reported that stress-responsive elements (STRE) mediate transcriptional regulation of the trehalose synthase genes *TPS1*, *TPS2*, and *TPS3* as well as the glycerol 3-phosphate dehydrogenase genes *GPD1* and *GPD2* [52, 53]. Msn2 and Msn4, which bind specifically to STRE-containing oligonucleotides [54], are controlled by the HOG-MAPK pathway [55]. Hence, we speculated that the accumulation of trehalose and glycerol by *MAL62* overexpression may start with the activation of the HOG-MAPK pathway, after which the trehalose synthase genes and glycerol 3-phosphate dehydrogenase genes are upregulated through STRE-mediated transcriptional regulation. This may explain our previous speculation that the enhancement in freezing tolerance by *MAL62* overexpression may involve multiple pathways [16]. A possible relationship between maltose metabolism and cryoprotectant synthesis is illustrated in Fig. 6.

Compared with the B-T and B-T + M strains, the BY14a strain has a higher intracellular trehalose content, which results in a higher cell viability at the onset of prefermentation (Fig. 5a, b). The B-T strain, which lacks trehalose, had a lower cell viability in the beginning, and the viability decreased rapidly. The B-T + M strain, which had a higher glycerol level, showed a smaller decrease in cell viability in the later stage of prefermentation (Fig. 5a, b). This phenomenon suggests that the high glycerol content induced by *MAL62* overexpression has some positive effects on the freezing tolerance. Due to the fact that trehalose and glycerol are the primary compounds affecting the freezing tolerance [38], the differences in trehalose and glycerol accumulation may play an important role in the different cell viabilities exhibited by the three strains after prefermentation and freezing for 7 days. It is possible that the increased level of glycerol and other gene expression changes caused by *MAL62* overexpression may play a role in the enhanced cell viability and leavening ability. Studies are underway in our laboratory to further confirm the roles of glycerol and other related genes in yeast cryoprotection.

Hyperosmotic stress, which causes desiccation and electrolyte release from yeast cells, is a major factor for loss of leavening activity after freezing and thawing [39]. Besides, the formation of ice crystals during freezing causes damage to the cell membrane and subcellular structure [56]. Previous studies have reported that fermentation with high glycerol-producing strains can result in improved cell viability and gas retention in dough [57]. Consistent with the report, our results showed that the B-T + M strain exhibits a higher glycerol content as well as improved gas retention in the dough, especially after the storage of the frozen dough for more than 1 week. We believe that this is partially because the HOG pathway and glycerol synthesis were activated by *MAL62* overexpression. It is worth noting that the B-T + M strain showed a high level of CO_2_ production during the 4 weeks of frozen dough storage. A possible reason is that *MAL62* overexpression enhances maltose metabolism, which is vital for dough fermentation [15].

## Conclusion

This study indicates that *MAL62* overexpression induces cryoprotection by the increased level of trehalose via the UDPG-dependent trehalose synthesis pathway. The increased glycerol content by *MAL62* overexpression also plays an important role in the cryoprotection, especially in the later stage of prefermentation. We believe that these findings shed new light on the mechanism of yeast response to freezing in lean bread dough and can help to improve industrial yeast strains.

## Supplementary information

**Additional file 1: Figure S1.** The starch and sucrose metabolism pathway was significantly increased.

**Additional file 2: Figure S2.** The glycerolipid metabolism pathway was significantly increased.

**Additional file 3: Figure S3.** The glycerophospholipid metabolism pathway was significantly increased.
