# Supplementary figures and images for: MAL62 overexpression enhances uridine diphosphoglucose-dependent trehalose synthesis and glycerol metabolism for cryoprotection of baker’s yeast in lean dough
Source: Microb Cell Fact. 2020 Oct 19;19:196. doi: 10.1186/s12934-020-01454-6 (PMC7574194; doi:10.1186/s12934-020-01454-6)

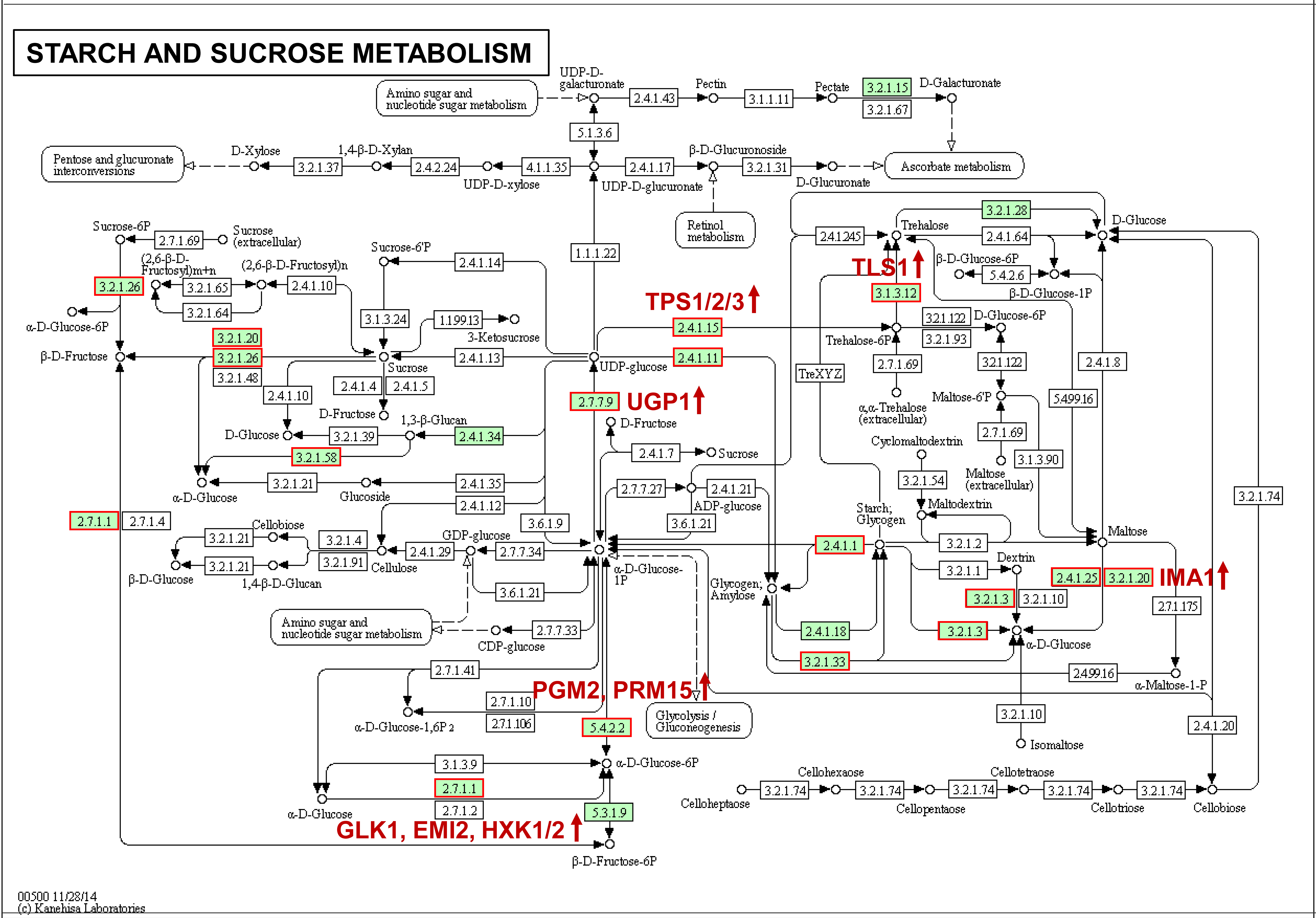

Supplement: Supplementary file 1 — Additional file 1: Figure S1. The starch and sucrose metabolism pathway was significantly increased. [file 12934_2020_1454_MOESM1_ESM.tif]

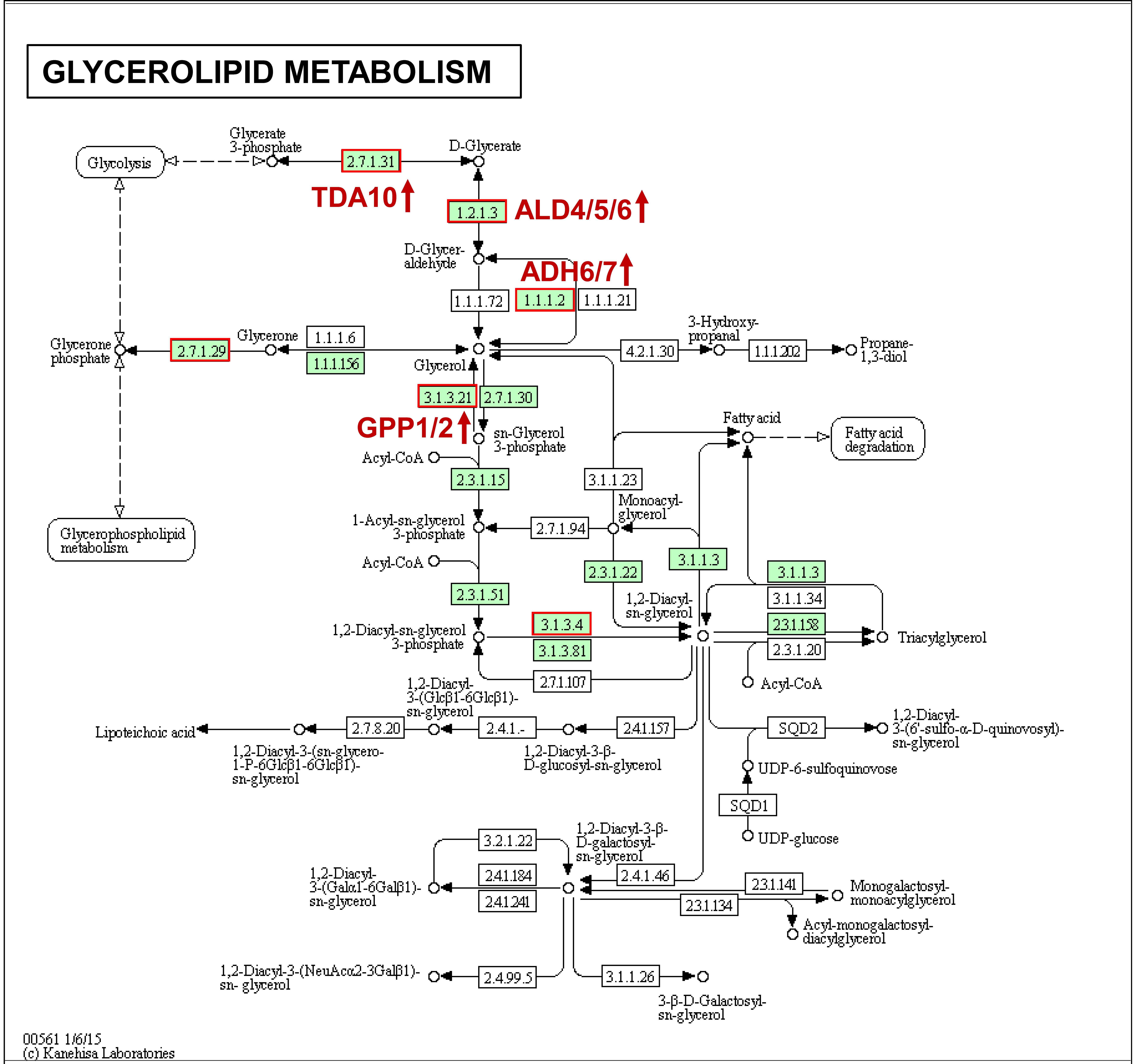

Supplement: Supplementary file 2 — Additional file 2: Figure S2. The glycerolipid metabolism pathway was significantly increased. [file 12934_2020_1454_MOESM2_ESM.tif]

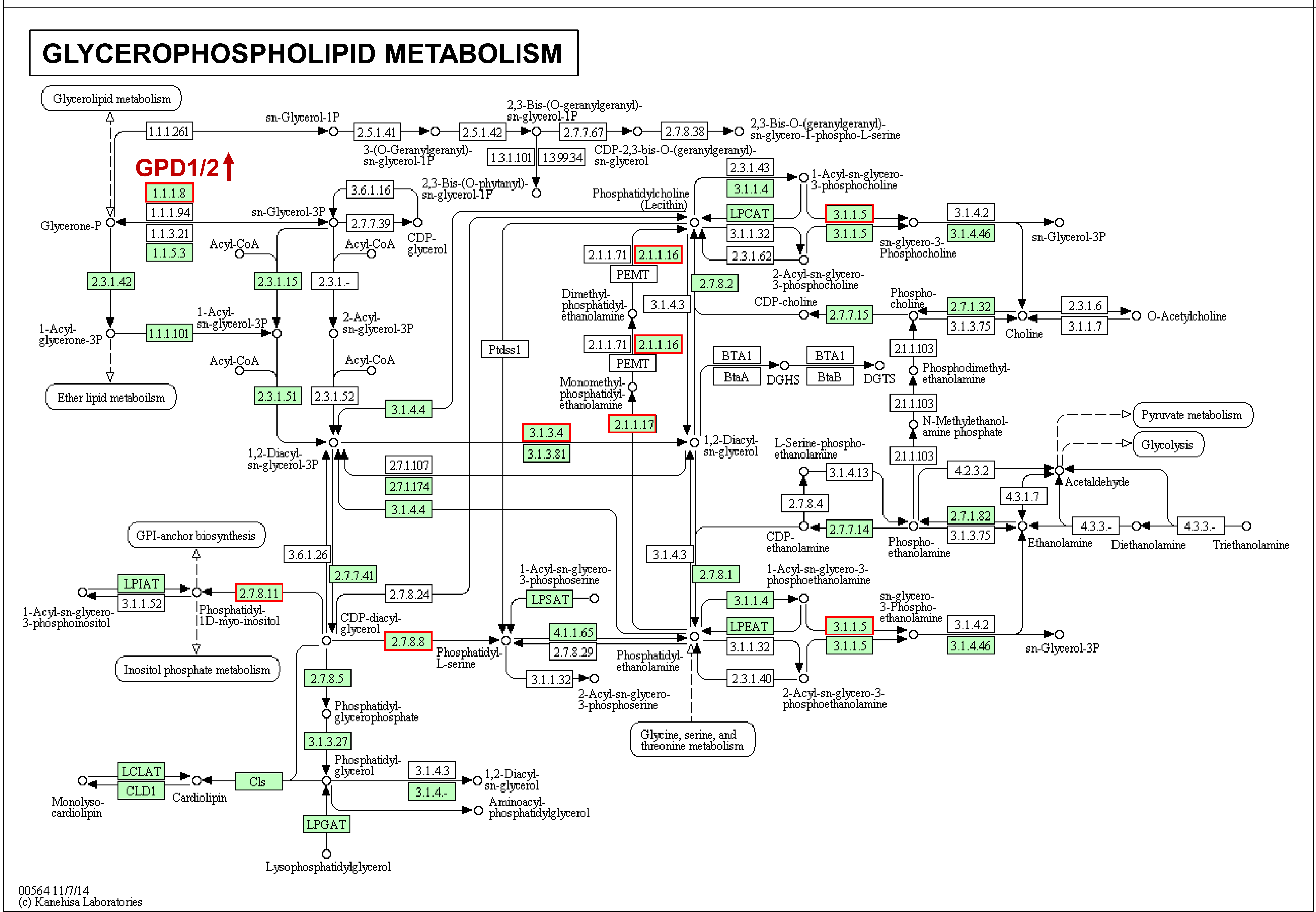

Supplement: Supplementary file 3 — Additional file 3: Figure S3. The glycerophospholipid metabolism pathway was significantly increased. [file 12934_2020_1454_MOESM3_ESM.tif]
